# Quantifying intracortical bone microstructure: A critical appraisal of 2D and 3D approaches for assessing vascular canals and osteocyte lacunae

**DOI:** 10.1111/joa.13325

**Published:** 2020-10-08

**Authors:** Katherine A. Williams, Neil J. Gostling, Joshua W. Steer, Richard O. C. Oreffo, Philipp Schneider

**Affiliations:** ^1^ Bioengineering Science Research Group Faculty of Engineering and Physical Sciences University of Southampton Southampton United Kingdom; ^2^ School of Biological Sciences Faculty of Environmental and Biological Sciences University of Southampton Southampton United Kingdom; ^3^ Bone and Joint Research Group Centre for Human Development Stem Cells and Regeneration Institute of Developmental Sciences Faculty of Medicine University of Southampton Southampton United Kingdom

**Keywords:** bone, fossil, histology, image analysis, osteocyte, vascular canal, X‐ray CT

## Abstract

Describing and quantifying vascular canal orientation and volume of osteocyte lacunae in bone is important in studies of bone growth, mechanics, health and disease. It is also an important element in analysing fossil bone in palaeohistology, key to understanding the growth, life and death of extinct animals. Often, bone microstructure is studied using two‐dimensional (2D) sections, and three‐dimensional (3D) shape and orientation of structures are estimated by modelling the structures using idealised geometries based on information from their cross sections. However, these methods rely on structures meeting strict geometric assumptions. Recently, 3D methods have been proposed which could provide a more accurate and robust approach to bone histology, but these have not been tested in direct comparison with their 2D counterparts in terms of accuracy and sensitivity to deviations from model assumptions. We compared 2D and 3D methodologies for estimating key microstructural traits using a combination of experimental and idealised test data sets. We generated populations of cylinders (canals) and ellipsoids (osteocyte lacunae), varying the cross‐sectional aspect ratios of cylinders and orientation of ellipsoids to test sensitivity to deviations from cylindricality and longitudinal orientation, respectively. Using published methods, based on 2D sections and 3D data sets, we estimated cylinder orientation and ellipsoid volume. We applied the same methods to six CT data sets of duck cortical bone, using the full volumes for 3D measurements and single CT slices to represent 2D sections. Using in silico test data sets that did deviate from ideal cylinders and ellipsoids resulted in inaccurate estimates of cylinder or canal orientation, and reduced accuracy in estimates of ellipsoid and lacunar volume. These results highlight the importance of using appropriate 3D imaging and quantitative methods for quantifying volume and orientation of 3D structures and offer approaches to significantly enhance our understanding of bone physiology based on accurate measures for bone microstructures.

## 1 INTRODUCTION

Bone histology is part of the daily armoury in biomedical research and the study of bone histology in extant and fossil animals has led to substantial shifts in the way we view extinct species and the evolution of key biological traits such as growth rate, metabolic rate and locomotion. Additionally, the arrangement of vascular canals within the bone cortex and the spaces wherein bone cells reside, the osteocyte lacunae, can provide information about developmental age (Carter et al., 2013; Lai et al., 2015) and growth rate (Castanet et al., 2000; de Margerie et al., 2002, 2004), and how the bone may have been loaded during the animal's lifetime (Britz et al., 2012; De Margerie et al., 2005; van Oers et al., 2015). Furthermore, bone microstructure is influenced by body mass (Cubo et al., 2014), metabolic constraints (Cubo et al., 2014) and phylogeny (Legendre et al., 2014). Studying bone histology in extant animals to discover correlations between intracortical bone microstructure and a variety of biological traits, from growth rate to lifestyle, has therefore significantly enhanced our understanding of extinct animals and evolution (Chinsamy‐Turan & Hurum, 2005; Erickson, 2014; Erickson et al., 2009; Padian & Lamm, 2013).

However, much remains to be learned from bone histology analysis. In palaeontology, histological descriptions of new fossils are typically qualitative, placing observed tissues into a set of bone types (De Ricqles, 1975) and relating these to traits, such as ’rapid growth’ or occasionally more specific growth rates (de Margerie et al., 2002). Although well‐established, the categories used to define the bone types do not fully capture the continuous nature of spatial and temporal variation in bone structure (de Boef & Larsson, 2007) and quantitative approaches are crucial to standardisation and robust testing of hypotheses.

A number of studies have taken such a quantitative approach, testing how measures for (intracortical) bone microstructures relate to other biological traits, including the orientation and density of vascular canals or the volume, shape and number and volume density of osteocyte lacunae. For example, vascular canal orientation has been correlated with bone deposition rate (de Margerie et al., 2002, 2004; Lee & Simons, 2015) and direction of bone loading (de Margerie et al., 2005; Marelli & Simons, 2014; Simons & O'connor, 2012), while increased vascular density (both number and volume density) has been related to accelerated growth rate (Cubo et al., 2014; de Buffrenil et al., 2008). Osteocyte lacunar shape and volume have been linked with body mass (D'Emic & Benson, 2013), growth rate (D'Emic & Benson, 2013), while genome size (D'Emic & Benson, 2013; Organ et al., 2007, 2009) and lacunar number density have been correlated inversely with body mass (Stein & Werner, 2013) and positively with femoral growth rate (Cubo et al., 2012). Understanding the correlations between morphology and biological origins of those morphologies can help us to understand the biology of living organisms, and to inform interpretations of the biology of extinct organisms from fossilised bone.

The correlations identified by such histological studies highlight the potential that histological approaches hold, if the different factors influencing these relationships can be disentangled. However, results have not been consistent across studies for all factors. For example, a number of studies have found correlations between decreased growth rate and laminar vascular canal orientations (de Margerie et al., 2002, 2004, 2005; Pratt et al., 2018), while others have not found this relationship to be significant, and instead argue that vascular canal orientation is more closely related to the direction of bone loading. Additionally, not all observations have been reliably reproduced across different studies in different species (de Margerie et al., 2002, 2004; Pratt & Cooper, 2018). The variation in results between studies is likely to be, in part, due to real differences in the biology between species, and as yet undefined factors determining tissue structure. However, it is also possible that methodological problems limit the accuracy and reliability of results generated. Further elucidation of the relationships between form and function and/or developmental origin is contingent on accurate and reproducible histological analysis.

In general, histological descriptions are based on a single, or small number of transverse thin tissue sections. Therefore, estimation of the orientation, shape or volume of structures in their native three‐dimensional (3D) context, such as intracortical canals or osteocyte lacunae, is based on their shape observed in two‐dimensional (2D) cross sections by light microscopy and the subsequent underlying assumptions needed to extrapolate the 2D information into 3D. The use of 2D histological methodologies, based on a single or small number of thin sections for each tissue sample, is a potential source of inaccuracy when characterising structures in a quantitative fashion. Doubt has been cast on the suitability of traditional 2D approaches for estimating 3D traits. Such traits include the shape and orientation of canals (Hennig et al., 2015), volumes of the osteocyte lacunae (D'Emic & Benson, 2013) and connectivity of pores and other structures (Qu et al., 2015), both within (Cooper et al., 2011; Pratt & Cooper, 2017; Stein & Prondvai, 2014) and outside palaeobiology (Schneider et al., 2007). Similarly, outside the bone field entirely, researchers in other areas have noted the importance of understanding 3D structures, from the structure of bubbles in cake batter (Tan et al., 2016) to complex branching plant root systems (Iyer‐Pascuzzi et al., 2010). In addition, the small specimen volume sampled in a histological section may not be representative of the bone as a whole, leading to an overestimation of the variation between specimens. Thus, similar variation could potentially be present within a single bone at a different volume of interest within the same anatomical site and/or at different anatomical sites. Using traditional histological methods, this would only be apparent if multiple sections were taken from each bone, and, to date, this is not the standard approach.

Across histology and palaeobiology, there is increasing adoption of micro‐computed tomography (µCT) (Abel et al., 2012; Knoll et al., 2018; Sanchez et al., 2012, 2016) as a non‐invasive 3D imaging technique. However, to date, little work has focussed on quantitative estimation of morphology and validation of existing 2D morphometric methods using the additional information 3D imaging can provide, especially at the microstructural level. In particular, the accuracy of 2D and 3D approaches have not been fully scrutinised, despite findings that, for example, shape and orientation cannot be distinguished based on 2D sections (Hennig et al., 2015; Pratt et al., 2018). Some 2D methods to quantify 3D structures require fewer assumptions to be made (for example measuring bone porosity only requires that the section taken is representative of the bone in general, rather than fitting a geometric model), and may be more robust than others. Therefore, it is important to understand which 2D approaches can be used robustly to quantitatively characterise 3D structures, such as intracortical canals or osteocyte lacunae. Furthermore, it is important to recognise what assumptions must be satisfied for these approaches to enable quantitative morphometry to be employed, and how 2D approaches compare to 3D approaches.

In this study, 2D and 3D approaches to quantify vascular canals and osteocyte lacunae are critically appraised and evaluated. 2D and 3D methods for quantitative morphometry are tested for their accuracy and cross‐validated against each other using simplified in silico test data sets in 2D and 3D, to estimate measures of histological interest, namely canal orientation and osteocyte lacunar volume. In addition, the same 2D and 3D methods are tested in experimental bone data sets from 2D virtual thin‐slice histology and 3D µCT, in order to compare and determine the quantitative morphometric results derived from 2D and 3D data sets.

## 2 METHODS

The first aim of this study was to compare the accuracy of 2D and 3D methods for quantitative morphometry of basic measures of the intracortical canal network and osteocyte lacunae, the canal orientation and osteocyte lacunar volume and cross‐validate their outcomes, using idealised shapes as in silico test data sets. The test data sets were varied to represent typical biological structures and experimental imaging settings, including:

- cylinders with circular and non‐circular cross sections for vascular canals,
- tri‐axial ellipsoids in different 3D orientations for osteocyte lacunae and
- different nominal resolutions of the imaging technique

To examine the limitations of the different 2D and 3D methods employed to estimate vascular canal and osteocyte lacunar measures.

The second aim of this study was to apply the same 2D and 3D methods to estimate vascular canal and osteocyte lacunar measures in experimental bone data sets, derived from 2D thin‐slice histology and µCT, in order to compare and critically appraise their quantitative morphometric outcomes. Here, experimental data sets were used to compare 2D and 3D methods in a more realistic context, and to determine whether these could be derived reliably from 2D tissue sections.

The quantitative methods used in this study combine in silico tests of 2D and 3D methods for estimating the orientation of cylinders (in silico models of vascular canals) and the volume of ellipsoids (in silico models of osteocyte lacunae). For the experimental data sets from the domestic duck (*Anas platyrhynchos*) generated and examined in this study, the same measures were estimated. It is straightforward to obtain the extant domestic duck due to their commercial production, unlike fossil samples. While the actual arrangement of these intracortical structures is not essential for the current work, the key is that the used bone material contains both vascular canals (i.e., tubes) and osteocyte lacunae (i.e., ellipsoids). The results from this study are, therefore, applicable to bone studies ranging from human biomedical samples to those looking at the bones of tyrannosaurs, and beyond this, any study inferring 3D properties of structures from their 2D cross sections.

For estimating the orientation of vascular canals, de Boef and Larsson's method for estimating orientation based on ellipses fitted to the 2D cross sections of canals (de Boef & Larsson, 2007) is compared with Pratt’s method for skeletonising and measuring 3D canal data sets (Pratt & Cooper, 2017). For estimating osteocyte lacunar volume, three methods were compared: a method based on fitting an ellipse to a single 2D cross section, one based on fitting ellipses to cross sections in two perpendicular planes with the aim of better characterising the average shape of a population of osteocyte lacunae (D'Emic & Benson, 2013) and a 3D method based on fitting ellipsoids to segmented 3D osteocyte lacunae from CT images.

### 2.1 Materials

Three commercially reared domestic ducks were purchased frozen (Large commercial supermarket chain, UK; University of Southampton ERGO ethics approval number 27443). The commercial harvest age for ducks within the UK is approximately 7 weeks (DEFRA, 2012). At this age, the ducks are not adult, but have reached adult body mass (Montes et al., 2005). The right tibiotarsus and humerus of each duck were dissected and 2 mm × 10 mm longitudinal matchsticks of cortical bone were cut from the midshaft (Figure S1) using a slow‐speed saw (Buehler Isomet, Esslingen am Neckar, Germany). The samples were fixed in 4% paraformaldehyde (PFA) for at least 24 h and stored in 70% ethanol at 4°C. The sample were then mounted vertically (so that reconstructed CT slices represent transverse sections of the long bones).

Ethanol was removed immediately before synchrotron‐based computed tomographic (SR CT) imaging and the samples were covered with a plastic tube (1.5 ml microcentrifuge tube) to reduce sample drying.

### 2.2 Image acquisition and processing of experimental 3D image data sets of vascular canals and osteocyte lacunae

The mid‐diaphysis of the samples were scanned at beamline I13‐2 of Diamond Light Source (Harwell Science and Innovation Campus, Didcot, UK), using a pink beam with an average X‐ray energy of 20 keV. The voxel size was set to 0.8 μm. A 100 ms exposure per projection was chosen for 4001 projections over 180˚ per scan, at a sample‐to‐detector distance of 20 mm. CT scans were reconstructed using standard filtered backprojection then binned twice to a final voxel size of 1.6 μm to improve the signal‐to‐noise ratio. In addition, the stack was reduced from 1079 slices to 1000 slices by removing slices at the top and bottom of the stack which differed in image contrast from the rest of the stack. The final stack volume was 1280 x 1284 x 1000 voxels. 2D data sets were generated by selecting the mid‐slice (slice number 500) from the reconstructed 3D CT stack.

Intracortical pores were segmented using a custom segmentation workflow in the free, open‐source image processing package Fiji/ImageJ (Schindelin et al., 2012) (see supplementary information for details including an example slice in Figure S2). Osteocyte lacunae and vascular canals were separated by volume (for 3D data sets) and by area (for 2D slices). Intracortical microstructures with volumes of 50‐500 μm^3^ were considered to be osteocyte lacunae (D'Emic and Benson, 2013), while features with volumes >1000 μm^3^ were classified as vascular canals. Objects with volumes between 500 and 1000 μm^3^ were excluded as these were larger than the majority of published estimates for avian osteocyte lacunae (roughly 100‐500 μm^3^ in most birds (D'Emic & Benson, 2013)). In the data sets of the present study, these objects often represented several osteocyte lacunae close together, especially near deposition surfaces where osteocytes may not be mature, that could not be easily separated. Using traditional histology in recent bone material, these would be possible to separate since the nuclei could be stained, however, such stains are not visible when using X‐rays. In 2D, an appropriate area threshold that separated osteocyte lacunae and vascular canals was more difficult to obtain from literature due to variability in canal diameter, however, through an iterative approach, an appropriate value for separating vascular canals from osteocyte lacunae was found to be >38 μm^2^.

### 2.3 Generation of 2D and 3D in silico test image data sets of vascular canals and osteocyte lacunae

For the first aim, we investigated whether for 3D in silico test image data sets, published 2D and 3D methods for quantitative morphometry are able to reproduce set rotational angles, aspect ratios and volumes of idealised shapes. This is done in such a way that all geometric assumptions are met, including perfect cylindricality for canals (de Boef & Larsson, 2007) and predominant lacunar alignments and thin‐section directions for osteocyte lacunae (Stein & Werner, 2013), and where—unlike in real conditions—each shape can be sliced perfectly through its midpoint. The rationale behind this simplification is that any problems for quantitative morphometry of intracortical microstructures can be highlighted in a controlled manner (best‐case scenario). In experimental data sets (realistic scenario), where the above assumptions are not (fully) met and each shape is not cut through its midpoint, any identified problems for characterising 3D microstructures in a quantitative fashion will be amplified.

Vascular canals in bone tissue are described as being approximately cylindrical in shape (de Boef & Larsson, 2007) and consequently, canals were represented by cylinders. Initially, a highly simplified test case was used: a single cylinder with a diameter of 10 voxels, which is within the range of canal diameters found in real bone in our preliminary studies of 7‐week‐old duck long bones when imaged at an isotropic voxel size of 1.6 μm, for instance through µCT. The cylinder was generated using Simpleware ScanIP (Vers 2016.09‐SP1; Synopsis, Inc.), a commercial image processing and model generation software. The cylinder was defined as a surface model with length 400 units and diameter 10 units, with the midpoint of the model centred in a cubic spatial domain of 500 × 500 × 500 units. A random value between 0˚ and 90˚ was generated for each of the three rotations around the *y*‐, *x*‐, and *z*‐axis (in this order) through the centre of the object (Figure S3). The cylinder was, therefore, rotated randomly in space, though its midpoint remained in the centre of the spatial domain. This process was repeated 100 times to generate a total of 100 individual cylinder data sets, representing 100 different random rotations in 3D space (Figure S3).

Vascular canals are, in reality, not always perfectly cylindrical (Hennig et al., 2015). Therefore, the sensitivity of the methods to deviations from perfect cylindricality were tested. Elliptic cylinders were generated with cross‐sectional aspect ratios of 1:1.25, 1:1.5 and 1:3 (Figure S4), representing perfectly cylindrical vascular canals (1:1), slightly elliptic vascular canals (1:1.25), moderately elliptic vascular canals (1:1.5) and extremely elliptic vascular canals (1:3). A total of 100 elliptic cylinders were generated for each aspect ratio, and each cylinder was rotated randomly in 3D using the method described above.

Osteocyte lacunae are often described as tri‐axial ellipsoids, with ratios of axis lengths of approximately 3:2:1 (Bach‐Gansmo et al., 2015) and a range of typical volumes between 100 and 500 μm^3^ in most birds and more than 1000 μm^3^ in the ostrich (D'Emic & Benson, 2013). In this study, the shape was simplified to a prolate spheroid with axis ratios of 3:1:1. The set ellipsoid was defined as an ellipsoid with axis lengths of 15 × 5×5 μm^3^ (Mader et al., 2013), giving a set volume of 196 μm^3^, within the typical range of lacunar volumes for birds (D'Emic & Benson, 2013).

Previous work has shown that the number of voxels are important, for example for estimation of ellipsoid/lacunae orientation (Mader, 2013). Therefore, ellipsoids were generated at three different sizes, which represents the case to image the set volume of 196 μm^3^ at three different voxel sizes, appropriate for imaging intracortical microstructures in bone using CT: 1.6 μm (typical for lab‐based μCT scanner and for reasonably large samples of a few millimetres), 0.8 μm and 0.33 μm (achievable in synchrotron settings but near the limit for current lab‐based systems and the general limit for standard CT, given by the diffraction limit of visible light around ~200 μm).

Like the cylinders, a highly simplified scenario was used, with individual ellipsoids placed at the centre of their respective cubic spatial domains (in this case the domain measured 50 × 50 × 50 units). The nominal voxel size was set by changing the axis lengths so that, when converted to voxels, the size was representative of an osteocyte lacuna imaged at that voxel size. This means that for a nominal voxel size of 1.6 μm, the axis lengths were adjusted to 9.375 × 3.125 × 3.125 arbitrary units, for 0.8 μm, the axis lengths were adjusted to 18.75 × 6.25 × 6.25 arbitrary units, and for 0.33 μm, the axis lengths were adjusted to 45 × 15 × 15 arbitrary units.

In order to represent randomly oriented osteocyte lacunae found in woven bone (Stein & Prondvai, 2014), surface models were given a random value between 0˚ and 90˚ for rotations around the *x*‐, *y*‐, and *z*‐axis through the centre of the object (same steps as shown in Figure S3 for cylinders). To represent parallel‐fibred bone in another osteocyte lacunar in silico model set, each ellipsoid was given a random value between 0˚ and 22.5˚ for rotations around the *x*‐, *y*‐, and *z*‐axis through the centre of the object (Figure S5). For each case, 100 ellipsoids with random orientations in 3D space were generated.

**Figure 1:**
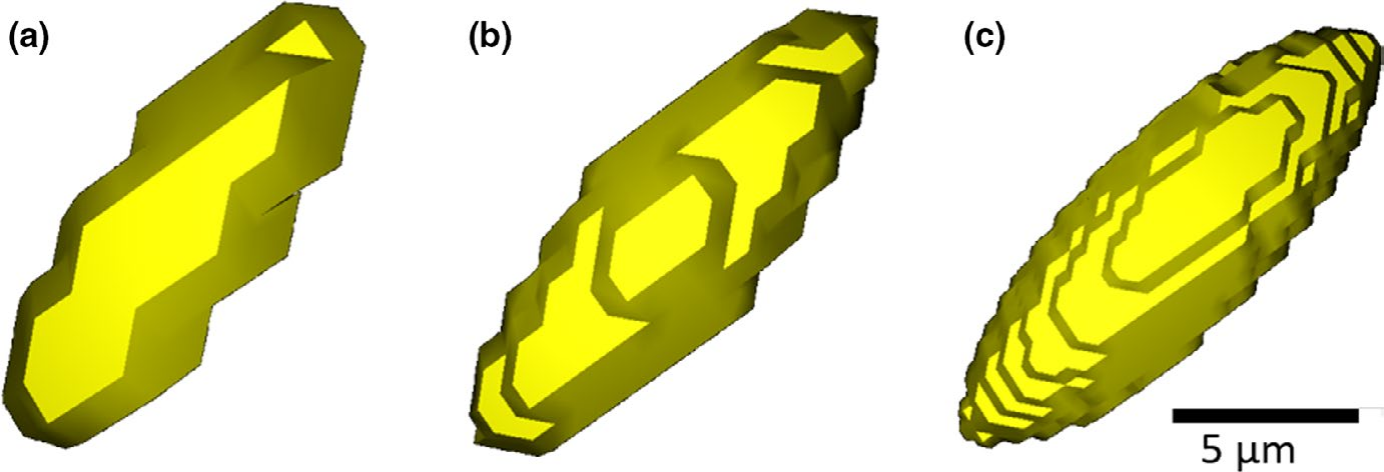
Osteocyte lacunar models at different voxel sizes. The ellipsoid shown measures 15 × 5 × 5 μm^3^. (a) At 1.6 μm voxel size, only a few voxels describe the ellipsoid (the major axis is approximately 9 voxels and the minor axis only 3 voxels long) and its shape is not well represented. (b) At 0.8 μm voxel size, the shape of the ellipsoid is better represented than at 1.6 μm voxel size (the major axis is approximately 19 voxels and the minor axis 6 voxels long). (c) At 0.33 μm voxel size, the shape of the ellipsoid is well represented by a larger number of voxels (the major axis is approximately 45 voxels and the minor axis 15 voxels long). (a‐c) Models were generated using ScanIP and visualised as surfaces using the 3D viewer in Fiji/ImageJ (Schindelin et al., 2012)

The surface models were converted to voxelised 3D images, using ScanIP’s ‘Surface to Mask’ tool. The resultant 3D image stacks were binarised in Fiji/ImageJ using an automated absolute thresholding method, namely the Li method based on minimising the cross‐entropy (Li & Tam, 1998). The effect of choosing a specific threshold value was not studied here but would affect the absolute volume estimated. For the ellipsoids, the different voxel sizes resulted in ellipsoid models with different numbers of voxels representing their volume (1.6 μm: ~48 voxels, 0.8 μm: ~383 voxels, 0.33 μm: ~5,301 voxels) as shown in Figure 1. Due to partial volume effects, not all the model lacunae were expected to have the same number of voxels: this would also be the case if identical lacunae were imaged in a real CT scan.

In order to compare estimates of lacuna volume based on 3D ellipsoid fitting and 2D 1‐plane and 2‐plane ellipse fitting methods, 2D slices were generated from the in silico 3D data sets. The mid‐slice of the data set was selected as the main slice because this cut directly through the midpoint of each object. In order to implement d’Emic and Benson's method to estimate osteocyte lacunar volume (D'Emic & Benson, 2013), a second slice is required, which is perpendicular to the first slice. Therefore, each osteocyte model was resliced in the *xz*‐plane, in order to create a second 2D image that passes through the midpoint of the object.

### 2.4 Quantification of vascular canals and osteocyte lacunae for 2D slices of 3D models

Vascular canal and osteocyte lacuna models were quantified using methods based on 3D volumes, and methods based on 2D sections, to estimate canal orientation and osteocyte lacunar volume.

**Figure 2:**
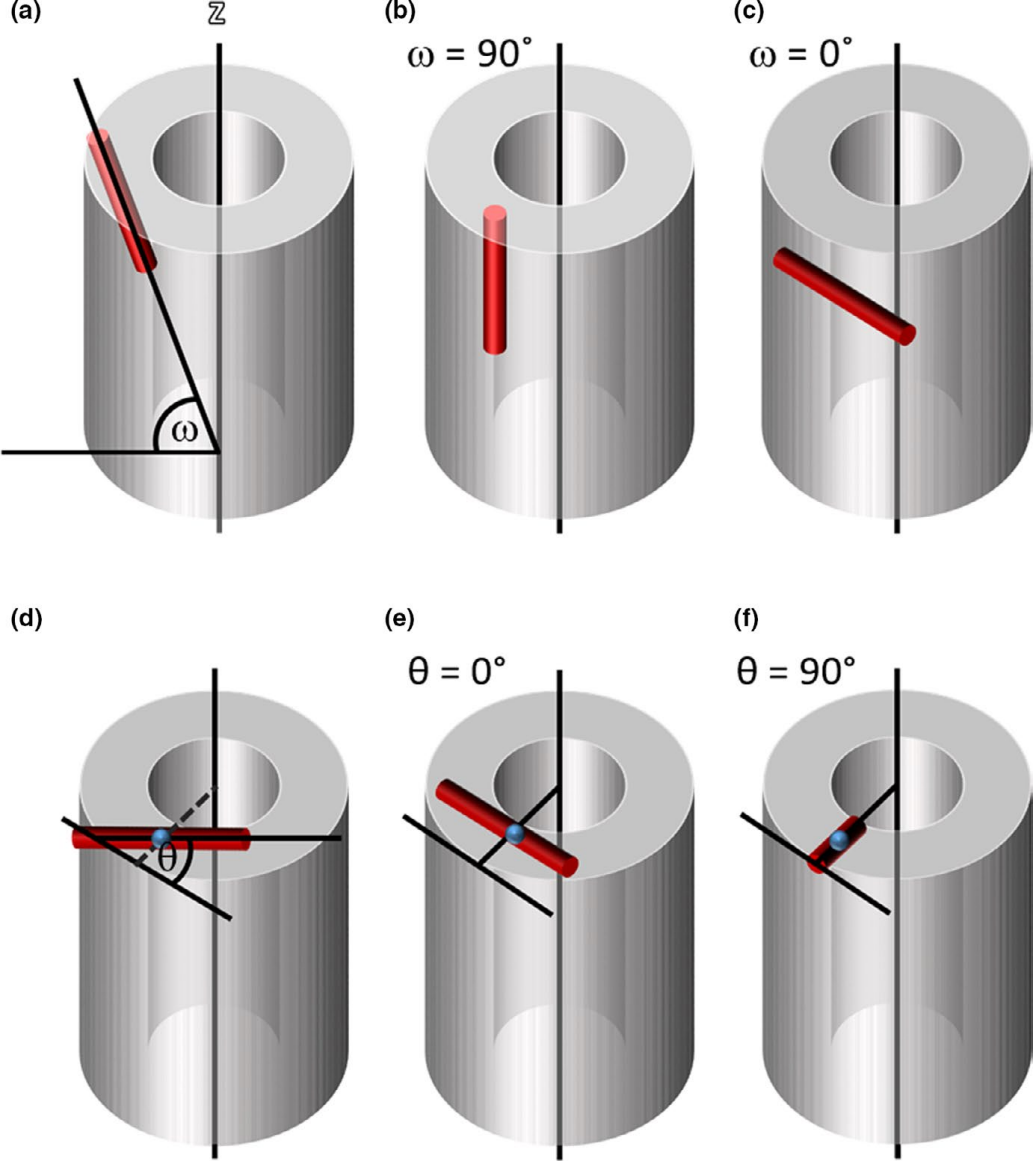
Longitudinal angle (ω) and radial angle (θ) definition, following de Boef and Larsson's definition (de Boef & Larsson, 2007). (a‐c) Longitudinal angle ω. (a) ω is defined as the angle between the main axis of the canal (red) and the longitudinal axis of the bone (z). (b) A longitudinal canal at ω = 90° and (c) ω = 0°. (d‐e) Radial angle θ. (d) θ is defined as the angle between the main axis of the canal and a tangent to the bone surface. (e) A canal at θ = 0° and (f) θ = 90°

The orientation of cylinders representing vascular canals was defined by two angles (Figure 2), a radial and longitudinal angle, following de Boef and Larsson's method (de Boef & Larsson, 2007). The longitudinal angle ω is the angle the canal makes relative to the longitudinal bone axis (0˚: perfectly transversal, 90˚: perfectly longitudinal), while the radial angle θ is a measure of whether the canal runs radially (90˚: like spokes on a bike wheel) or circumferentially (0˚: like the rim of a wheel).

**Figure 3:**
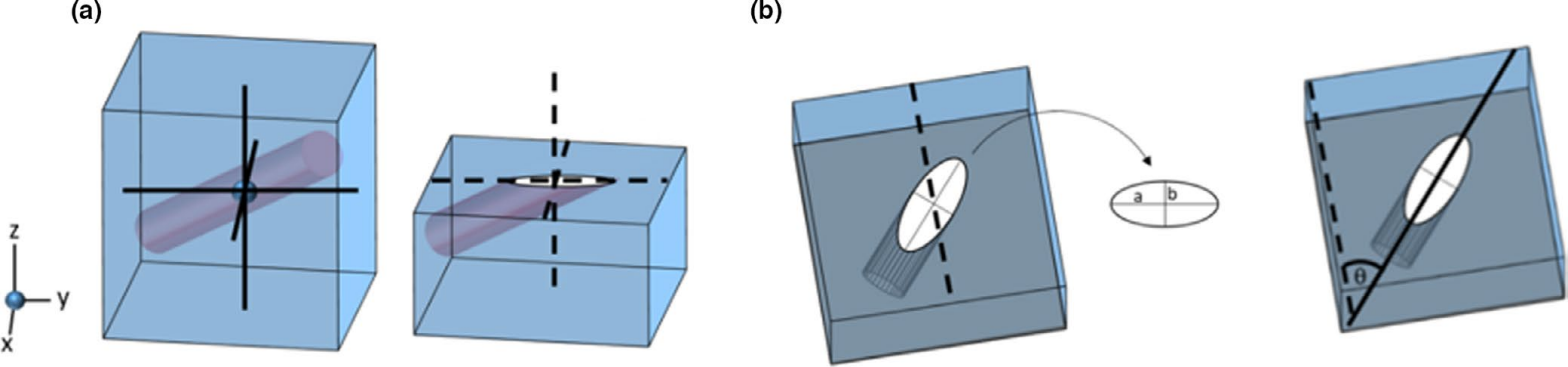
Estimation of longitudinal and radial angles of cylinders based on a 2D transverse section, following de Boef and Larsson's method (de Boef & Larsson, 2007). (a) A section through a cylinder is an ellipse. (b) The orientation of the major axis a of the ellipse is used to estimate the main orientation of the canal in the *xy*‐plane. The lengths of *a* and the minor axis of the ellipse *b* are used to estimate the longitudinal angle ω using trigonometry

In 2D, canal orientation was estimated by fitting best‐fit ellipses to the 2D canal cross section (Figure 3) using the ‘Analyze Particles’ tool in Fiji/ImageJ (de Boef & Larsson, 2007; Legendre et al., 2014). This method assumes that canals are circular in cross section. The longitudinal angle ω was calculated as

(1) $$\omega =\arcsin\left(\frac{b}{a}\right),$$

where *a* and *b* are the lengths of the major and minor axis of the best‐fit ellipse (Figure 3b).

The radial angle θ was estimated by calculating the angle between the major axis of the ellipse and a tangent to the edge of the bone surface. For in silico test data sets, the ‘tangent to the bone’ was fixed as a line parallel with the *x*‐axis (Figure 3b). For experimental data sets, the radial angle θ was estimated by calculating the angle between the major axis of the ellipse and the tangent to the bone surface nearest to the centroid of the ellipse (Figure 2d).

In 2D, osteocyte lacunae were quantified by fitting best‐fit ellipses to the segmented 2D cross sections (‘*xy*‐plane method’) using the ‘Analyze Particles’ tool in Fiji/ImageJ (de Boef & Larsson, 2007; Legendre et al., 2014). Mean lacuna volume ($\bar{Lc.V}$) was estimated by calculating the volumes of a prolate (i.e., elongated) spheroid:

(2) $$\bar{Lc.V}=\frac{\sum_{i=0}^{n}\left(\frac{4}{3}\pi r_{i,1}r_{i,2}^{2}\right)}{n},$$

with $r_{i,1}$ and $r_{i,2}$ being the major radius and minor radius of the individual best‐fit ellipse i and n the number of osteocyte lacunae. Additionally, a second estimate was made based on the work of D’Emic and Benson (D'Emic & Benson, 2013), using axes from two perpendicular 2D sections to capture the 3D shape more accurately, by estimating three different axes and thus, calculating the individual lacuna volumes based on a tri‐axial ellipsoidal shape:

(3) $$\bar{Lc.V}=\frac{\sum_{i=0}^{n}\sum_{i=0}^{n}\left(\frac{4}{3}\pi r_{i,1}r_{i,2}r_{i,3}\right)}{n},$$

with $r_{i,1}$ being the major radius length and $r_{i,2}$ and $r_{i,3}$ being the minor radii (‘*xy*/*xz*‐plane method’). $r_{i,2}$ and $r_{i,3}$ were estimated from the transverse section of the osteocyte lacuna, and $r_{i,1}$ from the major axis of an ellipse fitted to a perpendicular section following the assumption that osteocyte lacunae are on average aligned with the long axis of the bone.

### 2.5 Quantification of vascular canals and osteocyte lacunae of 3D models and experimental 3D CT data

The longitudinal and radial angles ω and θ of vascular canals were also estimated using the full 3D data sets for both in silico test data and experimental data.

**Figure 4:**
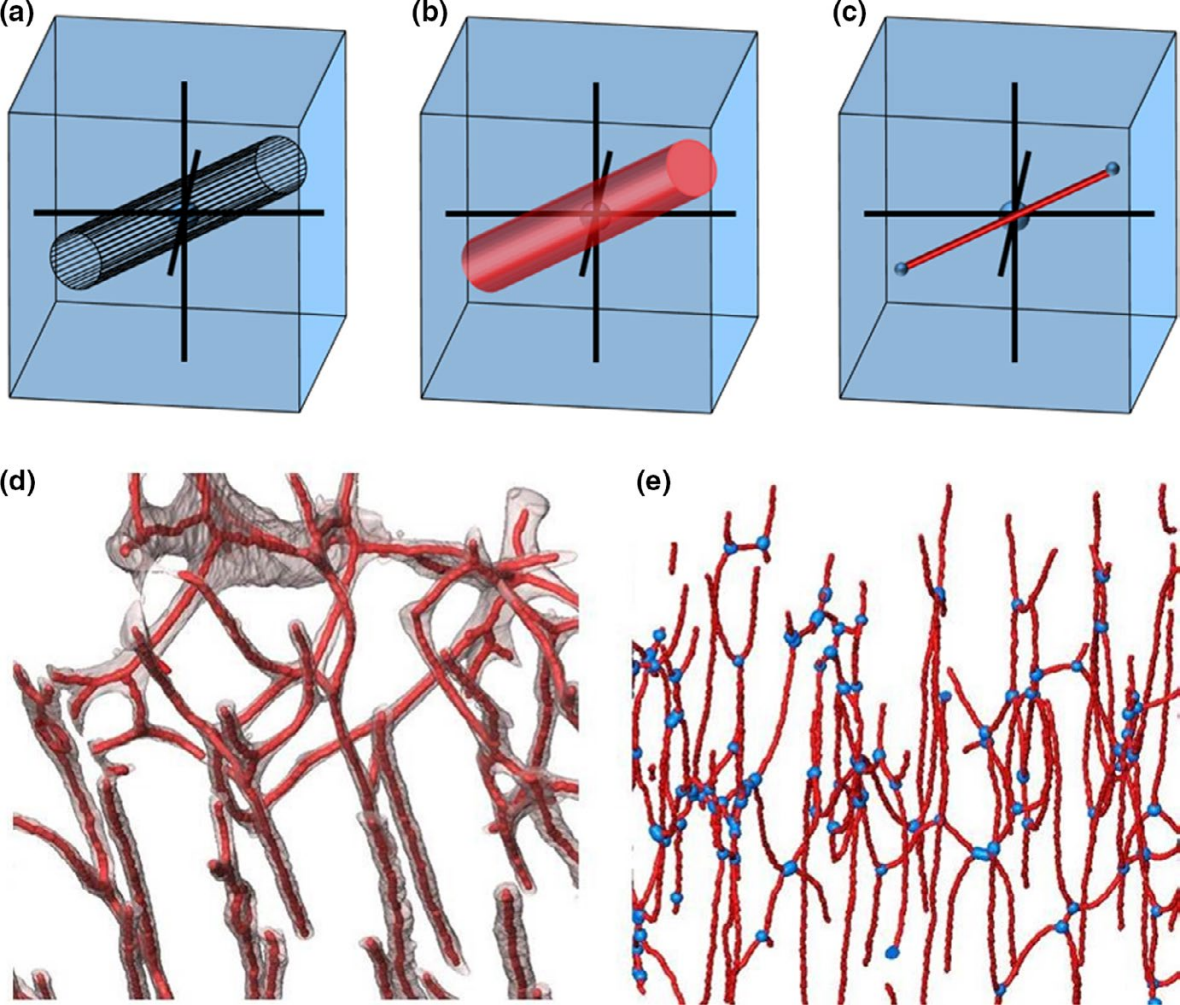
Quantification of vascular canals using 3D data sets. (a‐c) Processing test data sets. (a) Shape generated in 3D. (b) Shape voxelised and thresholded to create a binary voxel data set. (c) Voxel object thinned to a single line of voxels (skeleton) with the end points recorded (small blue spheres). (d‐e) 3D measurement of canal network from experimental data through skeletonisation process. (d) Skeletonisation: original segmented canals (transparent) are thinned to a single line of voxels (red, dilated for visualisation purposes). (e) Analysis: 3D locations of skeleton branch points (blue), length of branch and Euclidian distance between nodes are recorded. (d‐e) Visualisation of CT data sets performed in Avizo (version 9.3.0; Thermo Fisher Scientific)

The longitudinal and radial angles ω and θ of vascular canals were derived using a skeletonisation process (Figure 4) based on Pratt et al. (Pratt & Cooper, 2017; Pratt et al., 2018) and implemented using BoneJ (Doube et al., 2010), a plugin for ImageJ. A custom ImageJ macro was written to automate the process. First, the segmented canal was skeletonised using the ‘Skeletonize 2D/3D’ tool (Figure 4c‐d). Then, the skeleton was analysed using the ‘Analyze Skeleton’ tool, producing the coordinates of the end points of each branch in the skeleton (Figure 4e). Each canal was defined as the line between the nodes of the skeleton.

In Pratt's original method, each branch is broken into smaller segments to measure the orientations all the way along the branch, following its twists and turns. Here, a simpler skeleton has been used and each branch was defined as a straight line between the nodes. While this does reduce the accuracy of quantitative measures for curved canals, it provides an estimate of the overall direction of the canal. This simplification also reduces computational time, which can be significant in complex networks within large CT data sets. The longitudinal angle ω of a canal was estimated by calculating the angle between the skeleton branch (representing the canal) and the long axis of the bone. Additionally, the original method of Pratt et al. (Pratt & Cooper, 2017; Pratt et al., 2018) was adapted to estimate the longitudinal angle ω relative to the longitudinal bone axis rather than the reference system of the CT scan, taking into account bones that have not been mounted perfectly along the rotation axis of the CT setup (*z*‐axis). For experimental data sets, the scan was set up so that the long axis of the bone was (as closely as possible) aligned with the *z*‐axis of the CT scan. However, in some cases, the long axis of the bone was not perfectly aligned with the rotation axis and therefore, a correction was applied as follows: The orientation of the longitudinal bone axis for the experimental CT data was defined by fitting a circle to manually selected points on the surface of the bone in the first slice of the stack, and using a circle of the same diameter to fit the bone surface as closely as possible in the last slice of the stack. The bone axis was defined as the line between the centres of those two circles, and used as a correction when the longitudinal angle ω was calculated. For in silico test data sets, this was fixed as a line parallel to the *z*‐axis of the domain (Figure S6).

The radial angle θ was estimated by calculating the angle of the skeleton branch in the transverse plane relative to the nearest tangent to the bone surface at the *z*‐slice level of the mid‐point of the canal (Figure 2d).

The orientation of each canal estimated, using both the 2D and 3D method, was categorised as longitudinal (67.5˚ < ω ≤ 90˚), radial (0˚ < ω ≤ 67.5˚ and 67.5˚ < θ ≤ 90 ˚) or laminar (0˚ < ω ≤ 67.5˚ and 0˚ < θ ≤ 22.5˚) (Table 1), with oblique canals being excluded (0 < ω ≤ 67.5˚ and 22.5˚ < θ ≤ 67.5˚) following de Boef and Larsson (de Boef & Larsson, 2007). These major categories were used to calculate a longitudinal, radial and laminar index indicating the proportion or percentage of the canal network that falls into each of the major orientation categories (Table 1). For the 3D method, the overall length of the canal network in each category (as a proportion of the total network length including oblique canals) was used rather than the number of canals, in order to reduce the influence of short canals on the result and give a more accurate representation of the orientation of the whole vascular canal network. As oblique canals were excluded, (0 < ω ≤ 67.5˚ and 22.5˚ < θ ≤ 67.5˚) the three indices are not expected to add up to 1.

**Table 1:** Categories of major canal orientations

Orientation category	Longitudinal angle ω (degrees)	Radial angle θ (degrees)
Longitudinal	67.5‐90.0	0.0‐90.0
Radial	0.0‐67.5	67.5‐90.0
Laminar	0.0‐67.5	0.0‐22.5

Categories are based on canal's longitudinal angle ω and radial angle θ, following de Boef and Larsson (de Boef & Larsson, 2007).

In 3D, the volumes of the osteocyte lacunae were estimated by fitting best‐fit ellipsoids in 3D and using equation (2) (‘ellipsoid fit’ method) for the estimation of the mean lacuna volume. Ellipsoid fitting was carried out using the ‘Analyze Particles’ tool in the BoneJ (Doube et al., 2010) plugin for ImageJ.

### 2.6 Statistical analysis and visualisation

Statistical tests were carried out in IBM SPSS Statistics (version 25.0). For in silico test data sets Pearson's correlation coefficient was retrieved to characterise correlations between (ground‐truth) input dimensions of vascular canal and osteocyte lacuna in silico models compared to retrieved measures in 2D and 3D. A two‐tailed paired Student's *t*‐test was employed to test for significant differences (*p* < 0.05) between the random (but known) input angles of ω and θ and the estimated angles, in order to test whether the results differed significantly from those expected. For 2D and 3D results from experimental data sets, a two‐sample two‐tailed Student's *t*‐test was used to test for differences between the mean values of each measure from the 2D methods and the mean values of each measure from the 3D methods.

Bland‐Altman plots (also known as Tukey mean‐difference plots) were generated using the MatPlotLib package in the Anaconda distribution of Python 3.7. These plots are used to analyse the agreement between estimates or measures obtained by two different methods, by plotting their mean values along the *x*‐axis against their difference on the *y*‐axis. They allow different type of biases (e.g., fixed or proportional) between different methods to be identified by examining the error across different values for the measures. In other words, one can investigate whether and how the distinct methods differ over the range of possible values for those estimates or measures.

Visualisations were generated both in Avizo (version 9.3.0; Thermo Fisher Scientific), a commercial software package for segmentation, analysis and visualisation of 2D and 3D data sets, and in ImageJ’s 3D viewer.

## 3 RESULTS

### 3.1 Estimation of 3D canal orientation based on in silico 3D canal models

The ground‐truth input angles were compared with the angles estimated using the different methods. The accuracy of the estimate is described using the *r^2^* value (coefficient of determination), which indicates how well correlated the input and estimated values are, and Bland‐Altman plots which allow the differences in estimated values to be compared between the two methods and enable the errors to be explored further.

An implementation of Pratt’s method (Pratt & Cooper, 2017) was used to estimate the 3D orientation of each in silico vascular canal model based on 3D data as input. Skeletonisation of the vascular canals provided near perfect correlations between ground‐truth and estimated values for both the longitudinal angle ω (*r^2^* > 0.99) and radial angle θ (*r^2^* > 0.97) for all aspect ratios (Table 2), and 100% of canals were classified correctly as longitudinal, radial or laminar (Table 3).

**Table 2:** Sensitivity of 2D and 3D canal orientation estimates to aspect ratio of in silico 3D canals

	Aspect ratio of cylinder (set)	*r^2^* of estimated vs. set angle in 2D (mid‐slice)	Difference estimated vs. set angle (degrees) in 2D (mid‐slice)	*r^2^* of measured vs. set angle in 3D (skeleton)
Longitudinal angle ω	1:1	0.995	−0.3	1.000
	1:1.25	0.905	−6.1	1.000
	1:1.5	0.819	−10.3	1.000
	1:3	0.634	−14.2	1.000
Radial angle θ	1:1	0.986	0.4	1.000
	1:1.25	0.613	0.1	0.999
	1:1.5	0.376	−1.9	0.998
	1:3	0.253	7.4	0.978

The ground‐truth value for the set angles of in silico vascular canal models 3D is compared to the value that is estimated by fitting an ellipse to the transverse 2D mid‐slice and by skeletonising the canal in 3D.

**Table 3:** Effect of method for quantitative morphometry (2D or 3D) on accuracy of classification of vascular canals as longitudinal, radial or laminar

Aspect ratio	Category	Actual number of canals in category	Correctly classified canals in 3D	Correctly classified canals in 2D
1:1	Longitudinal	8	8 (100%)	7 (88%)
	Radial	16	16 (100%)	15 (94%)
	Laminar	19	19 (100%)	19 (100%)
1:1.25	Longitudinal	21	21 (100%)	0 (0%)
	Radial	17	17 (100%)	14 (82%)
	Laminar	19	19 (100%)	17 (90%)
1:1.5	Longitudinal	29	29 (100%)	0 (0%)
	Radial	20	20 (100%)	11 (55%)
	Laminar	12	12 (100%)	10 (83%)
1:3	Longitudinal	21	21 (100%)	0 (0%)
	Radial	19	19 (100%)	13 (68%)
	Laminar	16	16 (100%)	12 (75%)

### 3.2 Estimation of 3D canal orientation based on 2D slices of 3D canal models

**Figure 5:**
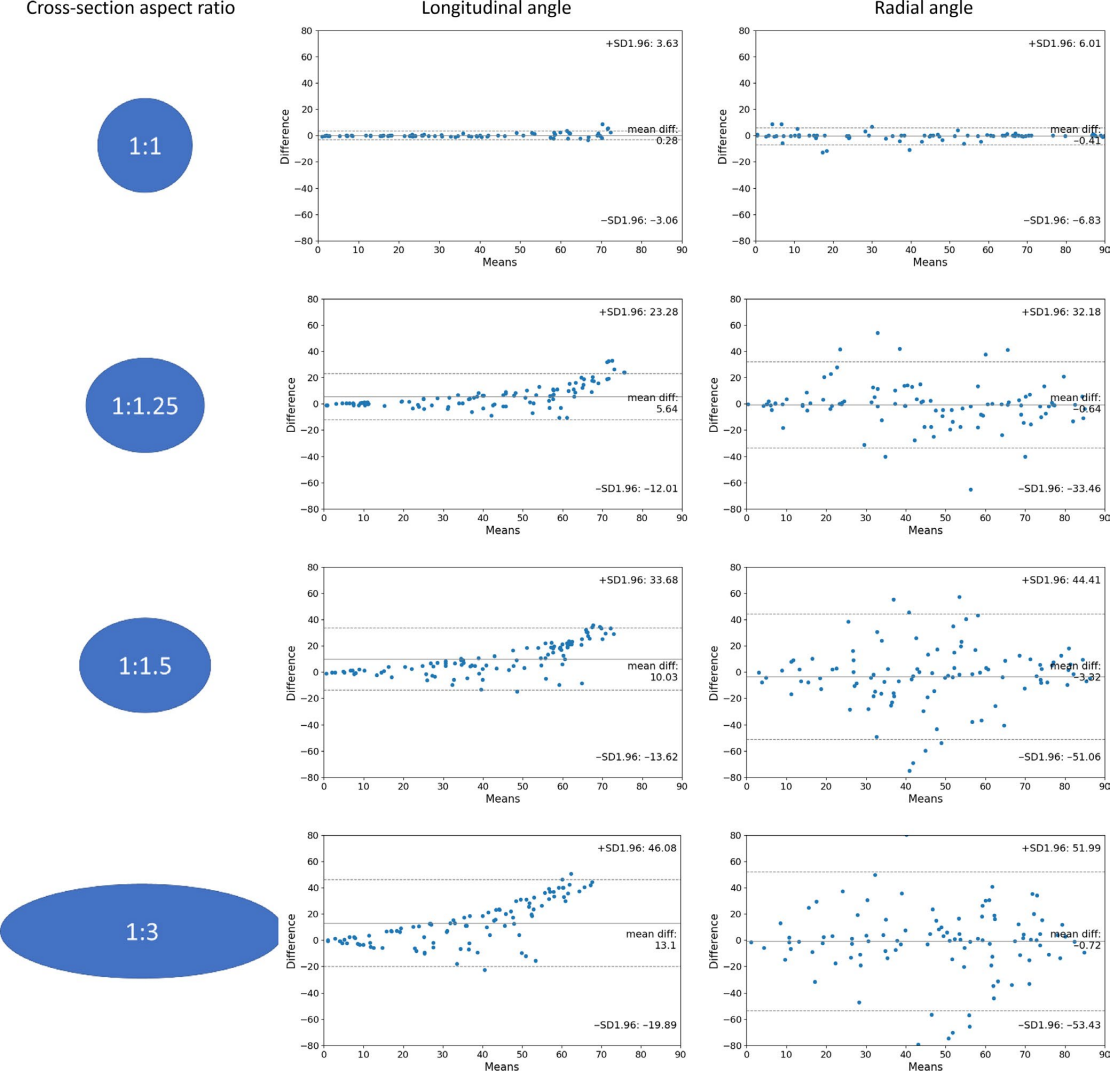
Sensitivity of 2D canal orientation estimates to aspect ratio of 3D canals. Bland‐Altman plots to show the agreement between estimated 3D angles (previously demonstrated to closely match the input angles) of in silico vascular canal models and angles derived from the transverse 2D mid‐slice, using cylinders in 3D with aspect ratios of 1:1, 1:1.25, 1:1.5 and 1:3 (*n* = 100 for each case), representing canals imaged at a voxel size of 1.6 µm. Means = mean of 2D and 3D measure. Difference = the difference between the 2D and 3D measures. Lines show mean difference and dotted lines show 95% confidence limits

The estimation methods of de Boef and Larsson (de Boef & Larsson, 2007) were used as a 2D comparison. When test shapes representing canals were perfectly cylindrical (cross‐sectional aspect ratio 1:1), 2D estimates of canal orientation using ellipse fitting to single cross sections correlated closely with set values for both the longitudinal angle ω (*r* ^2^ = 0.995) and the radial angle θ (*r* ^2^ = 0.968) (Figure 5 and Table 2), although longitudinal angle estimates became less accurate as the longitudinal angle increased. However, even a slightly elliptical shape of the cylinders (aspect ratio 1:1.25) reduced the correlation between set angles and 2D‐based estimates of both the longitudinal angle ω (*r* ^2^ = 0.905) and the radial angle θ (*r* ^2^ = 0.613), and more substantially for cylinders that have a more elliptical cross section (1:1.5, 1:3) (Table 2).

Hence, the aspect ratio of in silico vascular canal models was found to be critical for accurately estimating the radial angle θ. For a moderately elliptic vascular canal at an aspect ratio of 1:1.5, the coefficient of determination *r^2^* for the radial angle θ or in other words, the proportion of the explained variance in the radial angle that was retrieved from 2D data to estimate the input radial angle from 3D vascular canal models, dropped to below 40%. The reduction of the explained variance with increasingly elliptic aspect ratio for the longitudinal angle ω (Table 2) was smaller than for the radial angle θ. For a slightly elliptic vascular canal at an aspect ratio of 1:1.25, the explained variance was still above 90%, and above 80% at an aspect ratio of 1:1.5 (compared to less than 40% for the radial angle θ), while it was reduced to 63% for extremely elliptic vascular canals with an aspect ratio of 1:3. However, a systematic bias was observed in the estimation of the longitudinal angle ω, which was consistently underestimated with a mean difference in value of 5.64 degrees at an aspect ratio of 1:1.25, 10.03 degrees at 1:1.5, and 13.1 degrees at 1:3 (Figure 5). Looking at the Bland‐Altman plots, it is clear that this bias is more pronounced as the value of the longitudinal angle increases. On the contrary, no such bias was seen in the radial angle measurements, where, while spread out, the points were distributed symmetrically (Figure 5).

Although the *r^2^* values appear to demonstrate that the longitudinal angle ω is less severely affected by an elliptical aspect ratio than the radial angle θ, when these estimates are used to calculate the three indices (longitudinal, radial, laminar (de Boef & Larsson, 2007)), the opposite was the case. Even with a slightly elliptic aspect ratio (1:1.25), the use of the 2D method for quantitative morphometry did not result in any longitudinal angle estimates of more than 67.5˚ (Table 3), which is the defined threshold to classify canals as longitudinal (Table 1). Therefore, these canals would all be misclassified based on 2D data at hand, due to their deviation from perfect cylindricality (Table 3). For the radial and laminar categories, more canals were classified correctly. At 1:1, or at 1:1.25 cross‐sectional aspect ratio, more than 80% of the radial and laminar canals were classified correctly. However, the proportion of canals classified correctly decreased with increasing cross‐sectional aspect ratio. Furthermore, the error increases significantly with increasing longitudinal angles such that while cylinders in the plane of a section remained fairly accurately characterised in all cases, cylinders that were more aligned along the longitudinal bone axis were not appropriately represented by the estimated values for the longitudinal and radial angle (Figure 5).

### 3.3 Estimation of 3D canal orientation based on 2D slice vs. 3D volume of experimental 3D CT canal data

Following the results generated by vascular canal test data sets, experimental CT data sets were used (bone samples from right tibiotarsus and humerus of three 7‐week‐old domestic ducks) to test whether 2D estimations of canal orientation correlate with 3D estimations, assuming 3D estimates are accurate (as shown in the previous section).

**Figure 6:**
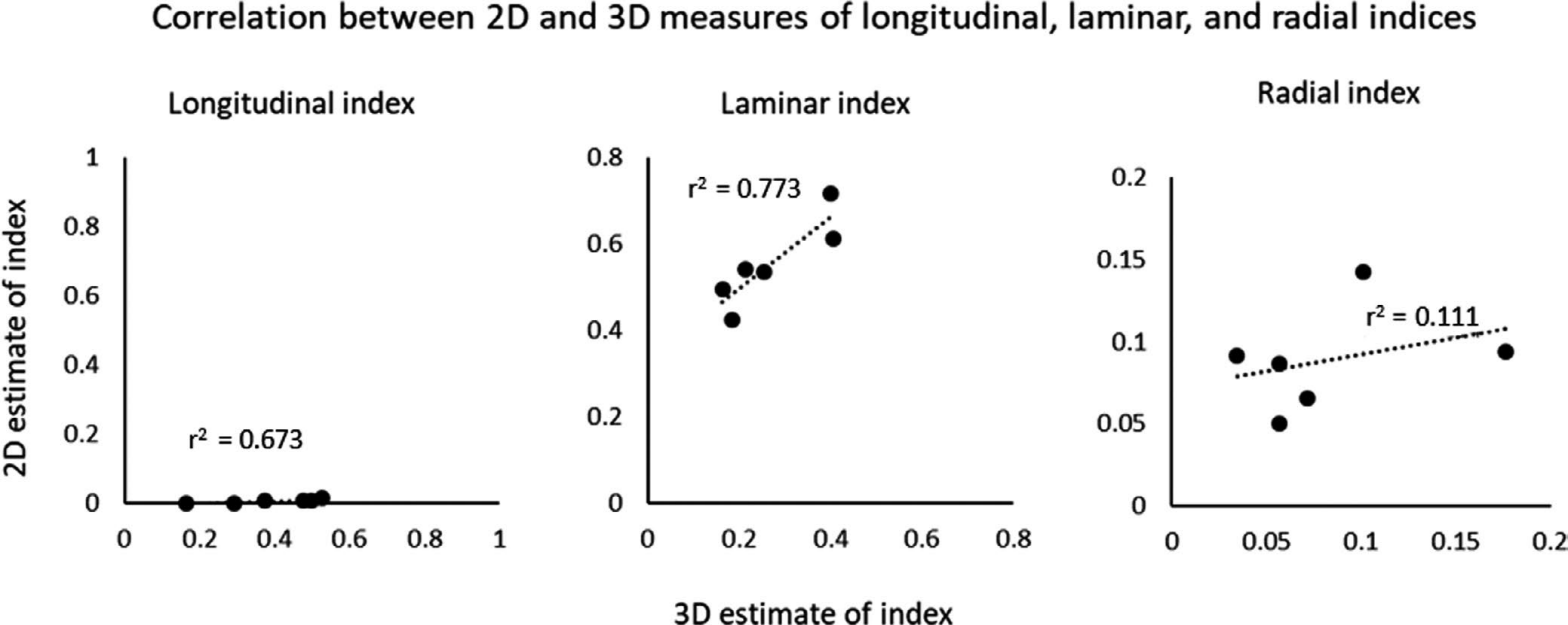
Correlations between 2D and 3D estimates for major orientation categories of vascular canals in experimental data. 2D estimates are based on the mid‐slice of each CT data set. No significant correlation was observed for the radial and longitudinal index. Data derived from 3D synchrotron‐based computed tomographic data sets from six duck bone samples

For the longitudinal, radial and a laminar index, specifying the proportion of canals in each of the major orientation categories (Table 1), no significant correlation (*p* > 0.05) was found between the indices calculated using 2D ellipse fits and those calculated using 3D skeletonisation (Figure 6). As predicted by the in silico test data sets, almost no canals were categorised as longitudinal based on 2D data using a threshold angle of 67.5˚, although the longitudinal index was between 0.16 and 0.53 according to estimates based on 3D data.

### 3.4 Estimation of osteocyte lacunar measures based on 2D slice vs. 3D volume of in silico 3D models

Volume measurements of osteocyte lacunae were carried out using different methods for idealised ellipsoids, using three different voxel sizes (0.33 μm, 0.8 μm, and 1.6 μm) and two different spatial arrangements representing woven bone and parallel‐fibred bone.

The first method tested for estimating mean lacuna volume ($\bar{Lc.V}$) of 3D ellipsoid lacuna models employing their major and minor radii from the transverse 2D mid‐slice of the 3D volume (*xy*‐plane method). Using this method, the mean lacuna volume was always underestimated, except at 1.6 μm voxel size (Tables 4 and 5). The volume was underestimated to a greater extent when the ellipsoids were aligned longitudinally, but the estimate's standard deviation was much reduced. Therefore, the results were more precise, but less accurate when ellipsoids were aligned longitudinally, as in parallel‐fibred bone.

**Table 4:** Estimations of osteocyte lacunar measures for woven bone in silico test data with randomly aligned osteocyte lacunae based on 2D slice vs. 3D volume

		Set value	1.6 μm		0.8 μm		0.33 μm	
			Mean	SD	Mean	SD	Mean	SD
2D	($\bar{Lc.V}$) *xy*‐plane method (μm^3^)	196	204	75	135	34	130	42
	($\bar{Lc.V}$) *xy*/*xz*‐plane method (μm^3^)	196	340	106	209	73	227	96
3D	($\bar{Lc.V}$) ellipsoid method (μm^3^)	196	311	49	201	18	195	6

Osteocyte lacunae have been modelled as ellipsoids, imaged at three different voxel sizes (1.6 μm, 0.8 μm, and 0.33 μm). For the 2D *xy*‐plane method, one transverse 2D mid‐slice of the 3D volume has been used to estimate mean lacuna volume ($\bar{Lc.V}$), while for the 2D *xy/xz*‐plane method, an additional perpendicular 2D slice has been included in the analysis. For the 3D ellipsoid method, the volume of best‐fitted ellipsoids has been used to calculate $\bar{Lc.V}$. SD: standard deviation.

**Table 5:** Estimations of osteocyte lacunar measures for parallel‐fibred bone in silico test data with longitudinally aligned osteocyte lacunae based on 2D slice vs. 3D volume

		Set value	1.6 μm		0.8 μm		0.33 μm	
			Mean	SD	Mean	SD	Mean	SD
2D	($\bar{Lc.V}$) *xy*‐plane method (μm^3^)	196	114	47	83	8	67	3
	($\bar{Lc.V}$) *xy*/*xz*‐plane method (μm^3^)	196	259	69	201	25	174	19
3D	($\bar{Lc.V}$) ellipsoid method (μm^3^)	196	307	63	231	18	204	3

Osteocyte lacunae have been modelled as ellipsoids, imaged at three different voxel sizes (1.6 μm, 0.8 μm, and 0.33 μm). For the 2D *xy*‐plane method, one transverse 2D mid‐slice of the 3D volume has been used to estimate mean lacuna volume ($\bar{Lc.V}$), while for the 2D *xy/xz*‐plane method, an additional perpendicular 2D slice has been included in the analysis. For the 3D ellipsoid method, the volume of best‐fitted ellipsoids has been used to calculate $\bar{Lc.V}$. SD: standard deviation.

The alternative method tested for estimating mean lacuna volume ($\bar{Lc.V}$) used two perpendicular mid‐sections (xy/xz‐plane method) to better capture the actual shape of the ellipsoids in 3D. When ellipsoids were randomly oriented, this measure slightly overestimated the volume (Table 4). The volume estimate was more accurate when ellipsoids were longitudinally aligned (Table 5) but still significantly different from the ground truth (one‐sample Student's *t*‐test, *p* < 0.01), even at the smallest voxel size of 0.33 μm (highest spatial resolution).

Finally, the ellipsoids were quantitatively characterised in 3D, by fitting an ellipsoid (ellipsoid method). At voxel sizes 0.8 μm or smaller, this method produced results that were close to the ground truth. At 0.33 μm voxel size, the standard deviation was low and the result was close to the expected value, although still significantly different to the expected value in the ‘parallel fibred’ case (one‐sample Student's *t*‐test, *p* < 0.01). However, the absolute difference in mean lacuna volume was small (8 μm^3^, or 4%).

The results observed in the current study are sensitive to voxel size. Although at the highest nominal resolution (0.33 μm voxel size), 3D measurements were relatively accurate, the same methods used were less accurate for larger voxel sizes. At 1.6 μm voxel size, the measured volume was approximately 30% larger than the ground‐truth value, due to partial volume effects and the choice of threshold used. Similar relationships between voxel size and estimated volume were also present for the estimates based on 2D methods for quantitative morphometry.

An additional note is that at 1.6 μm voxel size, too few voxels were sometimes present for reliable ellipsoid fitting, and therefore, not all ellipsoids could be fitted successfully (76% fitted successfully). The most accurate estimation for mean lacuna volume, close to the ground truth, was 3D ellipsoid fitting using high‐resolution data sets (Tables 4 and 5).

## 4 DISCUSSION

### 4.1 Vascular canal orientation

The results of this study show that the 2D method for estimating canal orientation published by de Boef and Larsson (de Boef and Larsson, 2007) is only effective as long as canals are perfectly cylindrical, and even then there is increasing error as the longitudinal index increases. For the idealised canals used in this study, a moderately elliptical shape of the canal (cross‐sectional aspect ratio 1:1.5) in 3D was sufficient to reduce significantly the proportion of the explained variance by the radial angle θ retrieved from 2D data to below 40%, while still more than 80% of the variance could be explained by the longitudinal angle ω when correlated to the ground truth at the same cross‐sectional aspect ratio of the respective canal. This high sensitivity of the radial angle θ to the aspect ratios of canals represents an important finding for application to histological sections. Thus, from a single cut section, it is not possible to tell whether a canal is elliptic or not, since that apparent shape may (also) relate to its radial orientation. Therefore, it is not possible to test whether the assumption of cylindricality has been met and whether the 2D method can be considered reliable. Even with approximately cylindrical canals (aspect ratio 1:1.25), the estimation of the radial angle θ was not robust (*r^2^* = 0.61) and, therefore, reduces the validity of any studies using radial orientations to estimate, for example, growth rates (Cubo et al., 2012; Legendre et al., 2013, 2014) or bone loading (De Margerie et al., 2005). Furthermore, the longitudinal angle ω was consistently underestimated using 2D methods, as also found in a previous study by Pratt and Cooper (Pratt and Cooper, 2018). Here, this underestimation was shown to lead to a high proportion of canals being wrongly categorised in terms of their 3D orientation. The results of any studies applying 2D methods for quantitative descriptions of intracortical canals should, therefore, be taken with caution, given their intrinsic limitations to derive 3D orientations based on 2D data. If multiple tissue sections were taken, it may be possible to identify reliably the 3D orientation of canals, however, this would require serial sectioning, which is both time‐consuming and destructive (Odgaard et al., 1990).

The 3D method published by Pratt et al. (Pratt & Cooper, 2017), on the contrary, was not sensitive to changes in cylinder cross‐sectional aspect ratio for the in silico vascular canal models, even when the aspect ratio was large (1:3). This method to estimate 3D canal orientation is, therefore, more robust to variation in canal shape. Additionally, since the 3D method is not dependent on estimating shape, it is expected to be less sensitive to lower spatial resolution than either the 2D methods tested here or any of the methods of characterising osteocyte lacunae. Moreover, as 3D visualisations of vascular canal skeletons demonstrated that skeletons and branch points were accurately identified by the method applied (Figure 4), 3D measurements of orientations in experimental data sets are expected to be accurate.

In biological data sets, shapes are seldom perfectly symmetrical or perfectly cylindrical. In the experimental data sets applied in this study, 3D visualisation suggests that canals are not perfectly circular in cross section (Figure 4). This result supports the findings of Pratt et al. (Pratt & Cooper, 2017), and is similar to data found in osteons (Hennig et al., 2015), which have been traditionally described as cylindrical in shape (de Boef & Larsson, 2007), but in fact are elliptic cylinders (Hennig et al., 2015).

Experimental CT data sets from young duck bone were used to compare the results derived when using 2D and 3D methods for quantitative morphometry to estimate canal orientation. The lack of correlation between 2D and 3D estimates of orientation indices suggests that appropriate assumptions for 2D estimation have not been satisfied. The poorest result was given for longitudinal canals, of which almost none have been classified as longitudinal in 2D, despite their presence being apparent from visualisations and 3D methods for quantitative morphometry. These findings supports previous work of Pratt et al. (Pratt & Cooper, 2018), who also demonstrated underestimation of longitudinal canals, as well as overestimation of laminar canals, which was also observed here (Figure 5).

It is possible that differences in vascular orientation between different limb bones in previous studies reflect some aspect of the organisation of intracortical bone microstructure. However, from individual tissue sections alone, it is impossible to determine whether this is truly reflecting orientation or whether shape is also important. While distinguishing between these two possible origins for the resulting difference in vascular orientation may not be necessary for simply recognising bone types (De Ricqles, 1975), the difference is important if the functional relationships between bone loading and microstructure and bone deposition and microstructure are to be understood, as the two different explanations may produce functionally different materials.

### 4.2 Osteocyte lacunar volume

Estimates of osteocyte lacunar size have been used in studies estimating genome size (Organ et al., 2007, 2009), growth rate and body mass between different species. Therefore, it is important that one can accurately estimate osteocyte lacunar volume to compare between species.

In the first 2D method tested (*xy*‐plane method) only the transverse mid‐slice is used to characterise the osteocyte lacuna and fit an ellipse to the cross section, where the lacuna is modelled as an ellipsoid. This method underestimated lacunar volume in all cases except for one scenario (1.6 μm voxel size). The method substantially underestimated lacunar volume when the lacunae were aligned longitudinally, since the transverse cross section cuts through the smallest axis of the lacuna. This result highlights the importance of being able to distinguish shape or size from orientation, as these osteocyte lacunae would appear much smaller if a transverse cut section was used compared to a longitudinal section.

The second method tested here (D'Emic & Benson, 2013) used two perpendicular sections to model the ellipsoid. Estimates of lacunar volume based on this method were closer to the ground truth. However, this second method must be applied with some caution. In real bone, not all osteocyte lacunae are likely to be aligned with the longitudinal bone axis since the lacunae tend to be aligned with collagen orientation (Kerschnitzki et al., 2011), which, in osteonal bone, runs circumferentially around osteons, and in woven bone is random, resulting in randomly oriented osteocyte lacunae. Therefore, this method can only be reliably applied to osteocyte lacunae in parallel‐fibred bone and should not be applied more broadly. In addition, for real samples, results for osteocyte volume would be less accurate than those observed here, because two sections could not capture the same osteocyte lacunae and therefore, only average measures of an osteocyte lacunae ensemble could be used. On the contrary, true 3D estimates of osteocyte lacunar volume were independent of orientation of osteocyte lacunar models, and are therefore more robust to differences in bone type and lacunar orientation. 3D methods for quantitative morphometry are, therefore, more accurate when estimating osteocyte lacunar volume.

Estimates for osteocyte lacunar volume varied between different voxel sizes, whether 2D or 3D methods for quantitative morphometry were used. Osteocyte lacunae with typical dimensions of 5‐20 µm fall close the limit of what can be assessed in terms of volume and especially shape when using lab‐based µCT and SR CT imaging. Although osteocyte lacunae can be resolved at slightly lower nominal resolutions than represented and performed here (voxel size >1.6 µm), the small number of voxels representing individual osteocyte lacunae make lacunar measures inaccurate. Firstly, the number of voxels is insufficient to accurately describe the shape of lacunae and identify their major and minor axes. Secondly, accuracy is decreased due to partial volume effects leading to errors in discretisation of the lacunar shape. Partial volume effects occur where pixels at the edge of the ellipsoid cover both object and background, and therefore, have an intermediate grey value, but must be classified as one or the other during (absolute) thresholding for image segmentation. Where few voxels are present, partial volume effects are stronger as edge voxels make up a greater proportion of the total object's volume. Partial volume effects also mean that slight changes in threshold values can have large effects on the measured volume of osteocyte lacunae.

For example, for typical dimensions of major and minor axes of osteocyte lacunae (mouse femur) in the order of 7.5‐10.0 μm and 2.5‐5.0 μm, respectively, (Mader et al., 2013) and an experimental voxel size of 0.5 μm, a difference of 1 voxel for one of the ellipsoid's axes (8.75 ± 0.5 μm, 5.0 ± 0.5 μm, and 2.5 ± 0.5 μm) can lead to changes in estimated volumes up to 20% when using Equation (2) (calculation not shown here) while similar problems exist for lacuna orientation as shown by Mader and colleagues (Mader, 2013). For a small and elongated osteocyte lacuna, modelled as prolate spheroid with axes ratios of 3:1:1 and major and minor axis lengths of 7.5 μm and 2.5 μm, respectively, a difference of 1 voxel for one of the ellipsoid's axes (7.5 ± 0.5 μm and 2.5 ± 0.5 μm) can lead to changes in estimated volume and aspect ratio of up to 21% and 43% when using Equation (3), respectively (calculation not shown here). Therefore, imaging microscopic objects such as osteocyte lacunae, even when using lab‐based μCT and SR CT at small voxel sizes around 1 μm and below, does not only limit the sample volume that can be assessed, but is also a regime where small variations in the input CT data, for instance due to partial volume effects or simply noise, limits the accuracy of morphometric measures that can be reasonably expected due to experimental limitations.

The effect of changing voxel size on accuracy of estimates of morphometric measures highlights the importance of selecting the appropriate nominal resolution for the feature of interest, especially for shape and volume. If osteocyte lacunae are quantitatively characterised using CT data sets with inappropriately large voxel sizes, volume estimates are likely to be highly inaccurate and variations large, making it impossible to compare confidently between different specimens. A sufficiently low voxel size (i.e., high nominal spatial resolution) is also important for quantifying vascular canals accurately, as larger voxel sizes may be sufficient to resolve the structures but not to reliably describe their shape, orientation or volume accurately. However, using the 3D skeletonisation method described in this work, it is only necessary to resolve the positions of the vascular canals and not their shape, in order to analyse their orientation and other aspects of the vascular network, making analysis of blood vessels less reliant on spatial resolution than that of osteocyte lacunae, even if they were of equivalent diameters. At the voxel sizes used here, the problem should be minimal, but at larger voxel sizes, such as 5‐15 μm, the ability to resolve canals has been found to degrade (Cooper et al., 2007).

Consequently, in order to accurately estimate osteocyte lacunar volume, 3D data sets should be used, at the highest spatial resolution possible. However, osteocyte number density counts should be possible for CT scans carried out at slightly lower nominal resolutions, as ellipsoid fitting was reliable for the 0.8 μm voxel size data sets in the current study, thus, making osteocyte number density counts possible using lab‐based μCT scanners.

### 4.3 Limitations

As a technical point for this study, it is worth noting that the random generation of input angles for this study failed to generate any values greater than 80 degrees for one of the data sets (cross‐sectional aspect ratio 1:1, longitudinal angle). This is an artefact of the random number generation process and not a result of the morphometric method failing to identify canals with a longitudinal angle of more than 80 degrees.

CT does have limitations, thus, in some cases and for certain questions, other approaches might be more desirable. For example, the high spatial resolutions required to accurately quantify osteocyte volume are not easy to achieve using lab‐based μCT scanners, and, if achieved, require typically long scan times (up to 12 h and more). For highest nominal resolutions, smaller samples are optimal due to the trade‐off between sample size and field of view covered for CT, and therefore, samples may still need to be cut for CT imaging. Using SR CT, high nominal resolutions can be achieved (around 1 μm and below) for short scan times (a few minutes), providing good image quality image data, yet, access to these SR facilities is limited, restricting their use as a standard tool easily accessible for many researchers. Yet, SR CT at high nominal resolutions can be necessary if very long scan times on state‐of‐the art lab‐based μCT are impractical or too expensive, in particular for highly powered studies with many samples. Additionally, some artefacts characteristic for CT data, such as beam hardening and ring artefacts, are not issues that would be present in traditional microscopy. Fortunately, given both the signal‐to‐noise and contrast‐to‐noise ratios were excellent in the current study using SR CT, segmentation of vascular canals and osteocyte lacunae was not effected by neither beam hardening nor ring artefacts (see Figure S2).

## 5 CONCLUSIONS

The findings of this study have important implications for the quantitative study of intracortical bone microstructure. There is a clear need for 3D imaging for the accurate estimation of canal orientation and osteocyte lacunar volume. Given the inaccuracies identified using idealised and best‐case‐scenario data sets in this study, applying the 2D methods for quantitative morphometry studied here to experimental 3D data sets will result in inaccurate results and, as a consequence, potentially incorrect conclusions.

3D estimates of morphometric measures for cylindrical and ellipsoidal objects, relevant to studying bone histology and many other fields from engineering to plant sciences, requires a truly 3D approach. Estimates based on 2D sections depend on strict geometric assumptions seldom met in biological and non‐biological samples, and therefore, measuring shape and orientation is often impossible from 2D data. Although the focus of the current study is on intracortical porosity in bone, the same shapes and problems are present in a wide range of structures, such as roots (Iyer‐Pascuzzi et al., 2010) or bubbles in batter (Tan et al., 2016), making the results here applicable and relevant far beyond bone.

Canal (cylinder) orientation cannot be estimated from 2D sections unless it can be shown that the assumption of perfect cylindricality is met, which is impossible from 2D cut sections alone. Therefore, any study aiming to include estimates of canal orientation should, if possible, not use estimates based on 2D ellipse fitting but prioritise 3D estimates based on high‐resolution 3D data, such as from high‐resolution μCT and SR CT scans.

The current study confirms that, if we are to understand how bone microstructure and biological traits are correlated, including measures that describe shapes, volumes and orientations, as well as connectivity, tortuosity, heterogeneity, it is crucial to carry out studies using truly 3D approaches, both for image acquisition as well as for methods employed to quantify the acquired 3D data. Whilst 2D imaging and evaluation has long been used as gold standard, it has clear limitations. However, even cutting‐edge lab based CT scanner are not always able to accurately characterise microstructures. Such analysis is at the forefront of scientific imaging and will become more accessible over time, thus, enabling researchers to access the high‐quality 3D methods over their limited 2D counterparts.

## AUTHOR CONTRIBUTIONS

KAW, ROCO, NJG and PS designed the study, KAW carried out the experiments and analyses, and KAW and PS wrote and revised the paper. JWS contributed to data analysis. All authors had editorial input on the manuscript and contributed to discussions throughout the study.

## Supporting information

Fig S1‐6

## References

[joa13325-bib-0001] Abel, R.L. , Laurini, C.R. & Richter, M. (2012) A palaeobiologist's guide to ‘virtual’ micro‐CT preparation. Palaeontologia Electronica, 15.

[joa13325-bib-0002] Bach‐Gansmo, F.L. , Weaver, J.C. , Jensen, M.H. , Leemreize, H. , Mader, K.S. , Stampanoni, M. et al. (2015) Osteocyte lacunar properties in rat cortical bone: differences between lamellar and central bone. Journal of structural biology, 191, 59–67. 26023043 10.1016/j.jsb.2015.05.005

[joa13325-bib-0003] Britz, H.M. , Carter, Y. , Jokihaara, J. , Leppanen, O.V. , Jarvinen, T.L.N. , Belev, G. et al. (2012) Prolonged unloading in growing rats reduces cortical osteocyte lacunar density and volume in the distal tibia. Bone, 51, 913–919. 23046687 10.1016/j.bone.2012.08.112

[joa13325-bib-0004] Carter, Y. , Thomas, C.D.L. , Clement, J.G. & Cooper, D.M.L. (2013) Femoral osteocyte lacunar density, volume and morphology in women across the lifespan. Journal of Structural Biology, 183, 519–526. 23872433 10.1016/j.jsb.2013.07.004

[joa13325-bib-0005] Castanet, J. , Rogers, K.C. , Cubo, J. & Boisard, J.J. (2000) Periosteal bone growth rates in extant ratites (ostriche and emu). Implications for assessing growth in dinosaurs. Comptes Rendus De L Academie Des Sciences Serie Iii‐Sciences De La Vie‐Life Sciences, 323, 543–550. 10.1016/s0764-4469(00)00181-5 10923210

[joa13325-bib-0006] Chinsamy‐Turan, A. & Hurum, J. (2005) Bone microstructure and growth patterns of early mammals. Journal of Vertebrate Paleontology, 25, 44a.

[joa13325-bib-0007] Cooper, D. , Erickson, B. , Peele, A. , Hannah, K. , Thomas, C. & Clement, J. (2011) Visualization of 3D osteon morphology by synchrotron radiation micro‐CT. Journal of Anatomy, 219, 481–489. 21644972 10.1111/j.1469-7580.2011.01398.x PMC3196753

[joa13325-bib-0008] Cooper, D. , Turinsky, A. , Sensen, C. & Hallgrimsson, B. (2007) Effect of voxel size on 3D micro‐CT analysis of cortical bone porosity. Calcified Tissue International, 80, 211–219. 17340226 10.1007/s00223-005-0274-6

[joa13325-bib-0009] Cubo, J. , Baudin, J. , Legendre, L. , Quilhac, A. & de Buffrenil, V. (2014) Geometric and metabolic constraints on bone vascular supply in diapsids. Biological Journal of the Linnean Society, 112, 668–677.

[joa13325-bib-0010] Cubo, J. , le Roy, N. , Martinez‐Maza, C. & Montes, L. (2012) Paleohistological estimation of bone growth rate in extinct archosaurs. Paleobiology, 38, 335–349.

[joa13325-bib-0011] de Boef, M. & Larsson, H.C.E. (2007) Bone microstructure: quantifying bone vascular orientation. Canadian Journal of Zoology‐Revue Canadienne De Zoologie, 85, 63–70.

[joa13325-bib-0012] de Buffrenil, V. , Houssaye, A. & Bohme, W. (2008) Bone vascular supply in monitor lizards (Squamata: Varanidae): Influence of size, growth, and phylogeny. Journal of Morphology, 269, 533–543. 18157866 10.1002/jmor.10604

[joa13325-bib-0013] de Margerie, E. , Cubo, J. & Castanet, J. (2002) Bone typology and growth rate: testing and quantifying ‘Amprino's rule’ in the mallard (Anas platyrhynchos). Comptes Rendus Biologies, 325, 221–230. 12017770 10.1016/s1631-0691(02)01429-4

[joa13325-bib-0014] de Margerie, E.D. , Robin, J.‐P. , Verrier, D. , Cubo, J. , Groscolas, R. & Castanet, J. (2004) Assessing a relationship between bone microstructure and growth rate: a fluorescent labelling study in the king penguin chick (Aptenodytes patagonicus). Journal of Experimental Biology, 207, 869–879. 10.1242/jeb.00841 14747417

[joa13325-bib-0015] de Margerie, E. , Sanchez, S. , Cubo, J. & Castanet, J. (2005) Torsional resistance as a principal component of the structural design of long bones: comparative multivariate evidence in birds. The Anatomical Record, 282, 49–66. 15584036 10.1002/ar.a.20141

[joa13325-bib-0016] Defra, U.K. (2012) An Overview of the UK Duck Industry. UK.

[joa13325-bib-0017] D'Emic, M.D. & Benson, R.B.J. (2013) Measurement, variation, and scaling of osteocyte lacunae: a case study in birds. Bone, 57, 300–310. 23954754 10.1016/j.bone.2013.08.010

[joa13325-bib-0018] Doube, M. , Kłosowski, M.M. , Arganda‐Carreras, I. , Cordelières, F.P. , Dougherty, R.P. , Jackson, J.S. et al. (2010) BoneJ: free and extensible bone image analysis in ImageJ. Bone, 47, 1076–1079. 20817052 10.1016/j.bone.2010.08.023 PMC3193171

[joa13325-bib-0019] Erickson, G.M. (2014) On dinosaur growth. Annual Review of Earth and Planetary Sciences, 42(1), 675–697.

[joa13325-bib-0020] Erickson, G.M. , Rauhut, O.W.M. , Zhou, Z.H. , Turner, A.H. , Inouye, B.D. , Hu, D.Y. et al. (2009) Was Dinosaurian Physiology Inherited by Birds? Reconciling Slow Growth in Archaeopteryx. PLoS One, 4. 10.1371/journal.pone.0007390 PMC2756958 19816582

[joa13325-bib-0021] Hennig, C. , Thomas, C.D.L. , Clement, J.G. & Cooper, D.M. (2015) Does 3D orientation account for variation in osteon morphology assessed by 2D histology? Journal of Anatomy, 227, 497–505. 26249538 10.1111/joa.12357 PMC4580107

[joa13325-bib-0022] Iyer‐Pascuzzi, A.S. , Symonova, O. , Mileyko, Y. , Hao, Y. , Belcher, H. , Harer, J. et al. (2010) Imaging and analysis platform for automatic phenotyping and trait ranking of plant root systems. Plant physiology, 152, 1148–1157. 20107024 10.1104/pp.109.150748 PMC2832248

[joa13325-bib-0023] Kerschnitzki, M. , Wagermaier, W. , Roschger, P. , Seto, J. , Shahar, R. , Duda, G.N. et al. (2011) The organization of the osteocyte network mirrors the extracellular matrix orientation in bone. Journal of Structural Biology, 173, 303–311. 21081167 10.1016/j.jsb.2010.11.014

[joa13325-bib-0024] Knoll, F. , Chiappe, L.M. , Sanchez, S. , Garwood, R.J. , Edwards, N.P. , Wogelius, R.A. et al. (2018) A diminutive perinate European Enantiornithes reveals an asynchronous ossification pattern in early birds. Nature Communications, 9, 937. 10.1038/s41467-018-03295-9 PMC5838198 29507288

[joa13325-bib-0025] Lai, X. , Price, C. , Modla, S. , Thompson, W.R. , Caplan, J. , Kirn‐Safran, C.B. et al. (2015) The dependences of osteocyte network on bone compartment, age, and disease. Bone Research, 3. 10.1038/boneres.2015.9 PMC4511381 26213632

[joa13325-bib-0026] Lee, A.H. & Simons, E.L.R. (2015) Wing bone laminarity is not an adaptation for torsional resistance in bats. Integrative and Comparative Biology, 55, E108. 10.7717/peerj.823 PMC4359045 25780775

[joa13325-bib-0027] Legendre, L.J. , Bourdon, E. , Scofield, R.P. , Tennyson, A.J.D. , Lamrous, H. , de Ricqles, A. et al. (2014) Bone histology, phylogeny, and palaeognathous birds (Aves: Palaeognathae). Biological Journal of the Linnean Society, 112, 688–700.

[joa13325-bib-0028] Legendre, L.J. , Segalen, L. & Cubo, J. (2013) Evidence for high bone growth rate in Euparkeria obtained using a new paleohistological inference model for the humerus. Journal of Vertebrate Paleontology, 33, 1343–1350.

[joa13325-bib-0029] Li, C.H. & Tam, P.K.S. (1998) An iterative algorithm for minimum cross entropy thresholding. Pattern Recognition Letters, 19, 771–776.

[joa13325-bib-0030] Mader, K. (2013) High‐throughput, synchrotron based tomographic microscopy tools for the quantitative characterization of complex structures: A bone and foam study. ETH Zurich.

[joa13325-bib-0031] Mader, K.S. , Schneider, P. , Müller, R. & Stampanoni, M. (2013) A quantitative framework for the 3D characterization of the osteocyte lacunar system. Bone, 57, 142–154. 23871748 10.1016/j.bone.2013.06.026

[joa13325-bib-0032] Marelli, C.A. & Simons, E.L. (2014) Microstructure and cross‐sectional shape of limb bones in Great Horned Owls and Red‐tailed Hawks: how do these features relate to differences in flight and hunting behavior? PLoS One, 9, e106094. 25162595 10.1371/journal.pone.0106094 PMC4146594

[joa13325-bib-0033] Montes, L. , de Margerie, E. , Castanet, J. , de Ricqlès, A. & Cubo, J. (2005) Relationship between bone growth rate and the thickness of calcified cartilage in the long bones of the Galloanserae (Aves). Journal of anatomy, 206, 445–452. 15857365 10.1111/j.1469-7580.2005.00410.x PMC1571507

[joa13325-bib-0034] Odgaard, A. , Andersen, K. , Melsen, F. & Gundersen, H.J.G. (1990) A direct method for fast three‐dimensional serial reconstruction. Journal of Microscopy, 159, 335–342. 2243366 10.1111/j.1365-2818.1990.tb03038.x

[joa13325-bib-0035] Organ, C.L. , Brusatte, S.L. & Stein, K. (2009) Sauropod dinosaurs evolved moderately sized genomes unrelated to body size. Proceedings of the Royal Society B‐Biological Sciences, 276, 4303–4308. 10.1098/rspb.2009.1343 PMC2817110 19793755

[joa13325-bib-0036] Organ, C.L. , Shedlock, A.M. , Meade, A. , Pagel, M. & Edwards, S.V. (2007) Origin of avian genome size and structure in non‐avian dinosaurs. Nature, 446, 180–184. 17344851 10.1038/nature05621

[joa13325-bib-0037] Padian, K. & Lamm, E.‐T. (2013) Bone histology of fossil tetrapods: advancing methods, analysis, and interpretation. Berkeley: University of California Press.

[joa13325-bib-0038] Pratt, I.V. & Cooper, D.M.L. (2017) A method for measuring the three‐dimensional orientation of cortical canals with implications for comparative analysis of bone microstructure in vertebrates. Micron, 92, 32–38. 27855318 10.1016/j.micron.2016.10.006

[joa13325-bib-0039] Pratt, I.V. & Cooper, D.M.L. (2018) The effect of growth rate on the three‐dimensional orientation of vascular canals in the cortical bone of broiler chickens. Journal of Anatomy, 233, 531–541. 30022496 10.1111/joa.12847 PMC6131975

[joa13325-bib-0040] Pratt, I.V. , Johnston, J.D. , Walker, E. & Cooper, D.M.L. (2018) Interpreting the three‐dimensional orientation of vascular canals and cross‐sectional geometry of cortical bone in birds and bats. Journal of Anatomy, 232, 931–942. 29520776 10.1111/joa.12803 PMC5979616

[joa13325-bib-0041] Qu, Q.M. , Blom, H. , Sanchez, S. & Ahlberg, P. (2015) Three‐dimensional virtual histology of silurian osteostracan scales revealed by synchrotron radiation microtomography. Journal of Morphology, 276, 873–888. 25809461 10.1002/jmor.20386

[joa13325-bib-0042] De Ricqles, A. (1975) Recherches paléohistologiques sur les os longs des tétrapodes VII. ‐ Sur la classification, la signification fonctionnelle et l’histoire des tissus oseux des tétrapodes. Première partie, structures. Annales de Paléontologie., 61, 78.

[joa13325-bib-0043] Sanchez, S. , Ahlberg, P.E. , Trinajstic, K.M. , Mirone, A. & Tafforeau, P. (2012) Three‐dimensional synchrotron virtual paleohistology: A new insight into the world of fossil bone microstructures. Microscopy and Microanalysis, 18, 1095–1105. 23026256 10.1017/S1431927612001079

[joa13325-bib-0044] Sanchez, S. , Tafforeau, P. , Clack, J.A. & Ahlberg, P.E. (2016) Life history of the stem tetrapod Acanthostega revealed by synchrotron microtomography. Nature, 537, 408. 27602519 10.1038/nature19354 PMC6485594

[joa13325-bib-0045] Schindelin, J. , Arganda‐Carreras, I. , Frise, E. , Kaynig, V. , Longair, M. , Pietzsch, T. et al. (2012) Fiji: an open‐source platform for biological‐image analysis. Nature methods, 9, 676–682. 22743772 10.1038/nmeth.2019 PMC3855844

[joa13325-bib-0046] Schneider, P. , Stauber, M. , Voide, R. , Stampanoni, M. , Donahue, L.R. & Müller, R. (2007) Ultrastructural properties in cortical bone vary greatly in two inbred strains of mice as assessed by synchrotron light based micro‐ and nano‐CT. Journal of Bone and Mineral Research, 22, 1557–1570. 17605631 10.1359/jbmr.070703

[joa13325-bib-0047] Simons, E.L. & O'Connor, P.M. (2012) Bone laminarity in the avian forelimb skeleton and its relationship to flight mode: testing functional interpretations. The Anatomical Record: Advances in Integrative Anatomy and Evolutionary Biology, 295, 386–396. 10.1002/ar.22402 22241723

[joa13325-bib-0048] Stein, K. & Prondvai, E. (2014) Rethinking the nature of fibrolamellar bone: an integrative biological revision of sauropod plexiform bone formation. Biological Reviews, 89, 24–47. 23647662 10.1111/brv.12041

[joa13325-bib-0049] Stein, K.W.H. & Werner, J. (2013) Preliminary analysis of osteocyte lacunar density in long bones of tetrapods: All measures are bigger in sauropod dinosaurs. PLoS One, 8(10), e77109. 24204748 10.1371/journal.pone.0077109 PMC3812986

[joa13325-bib-0050] Tan, M. , Chin, N. , Yusof, Y. & Abdullah, J. (2016) Novel 2D and 3D imaging of internal aerated structure of ultrasonically treated foams and cakes using X‐ray tomography and X‐ray microtomography. Journal of Food Engineering, 183, 9–15.

[joa13325-bib-0051] van Oers, R.F.M. , Wang, H. & Bacabac, R.G. (2015) Osteocyte Shape and Mechanical Loading. Current Osteoporosis Reports, 13, 61–66. 25663071 10.1007/s11914-015-0256-1 PMC4352610

