# Supplementary material for: Quantifying intracortical bone microstructure: A critical appraisal of 2D and 3D approaches for assessing vascular canals and osteocyte lacunae
Source: J Anat. 2020 Oct 8;238(3):653–68. doi: 10.1111/joa.13325 (PMC7855084; doi:10.1111/joa.13325)
Supplement: Supplementary file 1 — Fig S1‐6 [file JOA-238-653-s001.docx]

**Supplementary material**


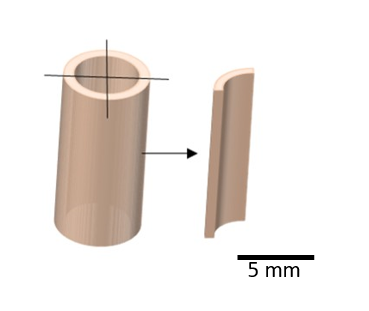


***Figure S1: Sectioning of duck specimens.*** *Bone diaphysis of duck tibiotarsus and humerus were sectioned longitudinally to create matchstick-shaped samples measuring approximately 2 mm* × *10 mm, and mounted vertically for CT imaging.*

**Image processing workflow**

Figure S2A shows a greyscale CT slice before processing, and Figure S2B shows the same slice after the segmentation workflow but before the vascular canals and osteocyte lacunae were separated. First, a 3D Gaussian filter with a standard deviation of 1 was applied to reduce image noise. Images were binarised using a minimum cross entropy thresholding algorithm (Li and Tam, 1998). The bone volume was separated from small image artefacts and noise by a single-voxel erosion and dilation, followed by a component labelling process to remove all except the largest object (*i.e.*, the cortical bone tissue), resulting in a cleaned dataset. The pores within the bone cortex were filled by dilation and erosion operations to create a solid cortical mask. This mask was used to define the edges of the canal network and to extract the intracortical pores (intracortical canals and osteocyte lacunae) by applying an AND function with the inverted cleaned dataset or in other words, by applying the mask to the inverted cleaned dataset that represents the intracortical pores, the medullary cavity and the space around the respective long bone.


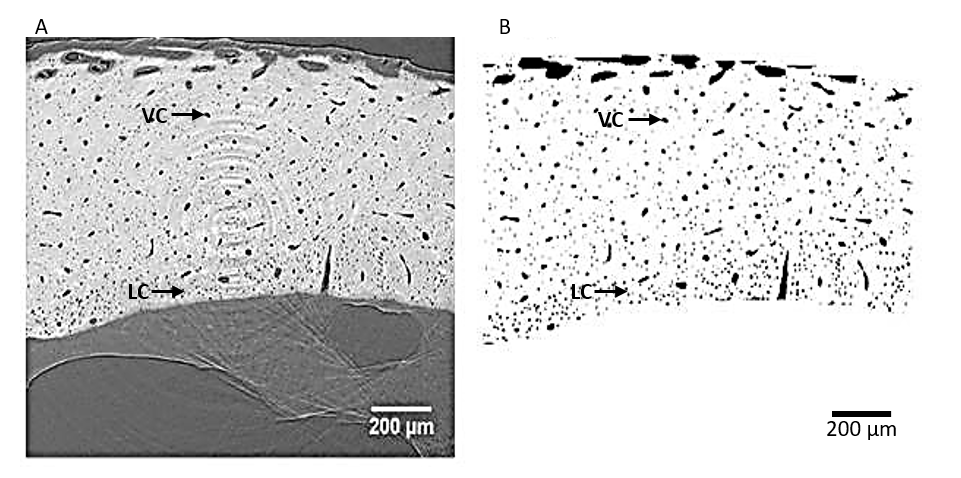


***Figure S2: Segmentation of duck CT datasets****. (A) CT slice showing a transverse section through bone from the tibiotarsus of a 7-week-old duck with vascular canals (VC) and osteocyte lacunae (LC). (B) Segmented vascular canals and osteocyte lacunae, later separated by volume (in 3D) and area (in 2D).*


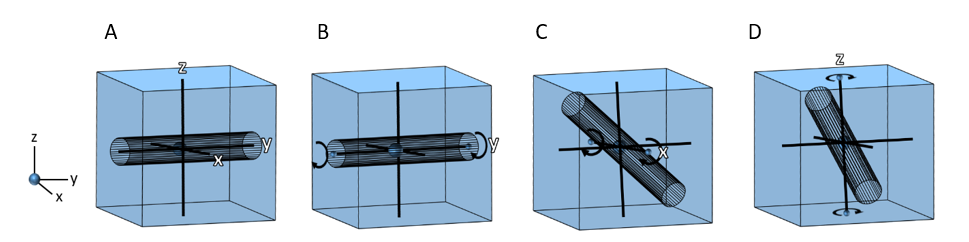


***Figure S3: Idealised vascular canals (cylinders) were generated as test datasets.*** *(A) Cylinder generated with long axis parallel to the y-axis of the domain and the centre of the cylinder in the centre of the domain. (B) Cylinder rotated about the y-axis (in the case of a perfect cylinder, this action does not change anything). (C) Cylinder rotated about the x-axis. (D) Cylinder rotated about the z-axis. (A-D) 100 cylinders were generated with this approach in separate cubic domains.*


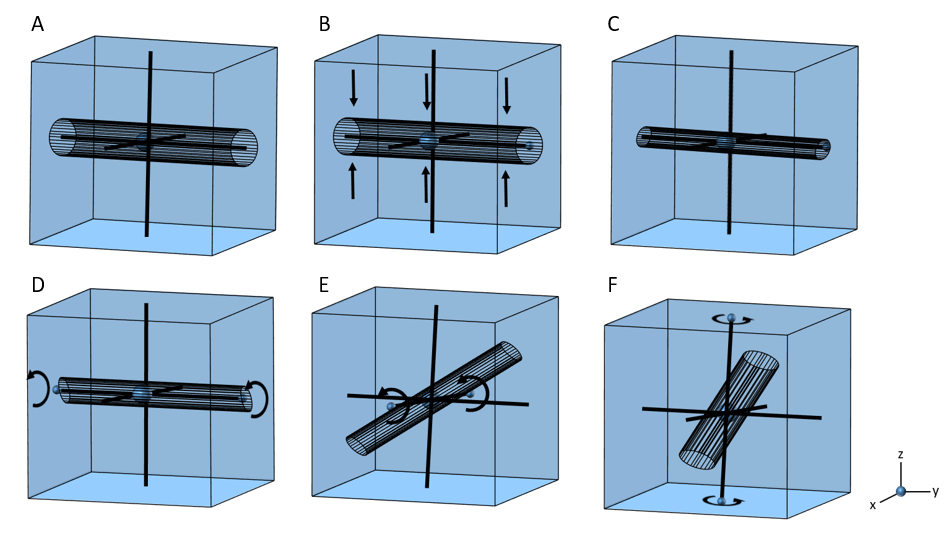


***Figure S4: Elliptic cylinders generated to test sensitivity of estimated spatial orientation to deviations from assumption of perfect cylindricality.*** *(A) Cylinder generated with long axis parallel to the y-axis of the domain and the centre of the cylinder in the centre of the domain. (B-C) Cylinder radially compressed in order to give a cross-sectional aspect ratio of 1:1.25 (slightly elliptic), 1:1.5 (moderately elliptic), or 1:3 (extremely elliptic) for the resulting elliptic cylinders. (D) Cylinder rotated about the y-axis (randomising the direction of the cross section’s major axis). (E) Cylinder rotated about the x-axis. (D) Cylinder rotated about the z-axis. (A-F) 100 cylinders were generated in this manner in separate cubic domains for each cross-sectional aspect ratio (in total 300 cylinders).*


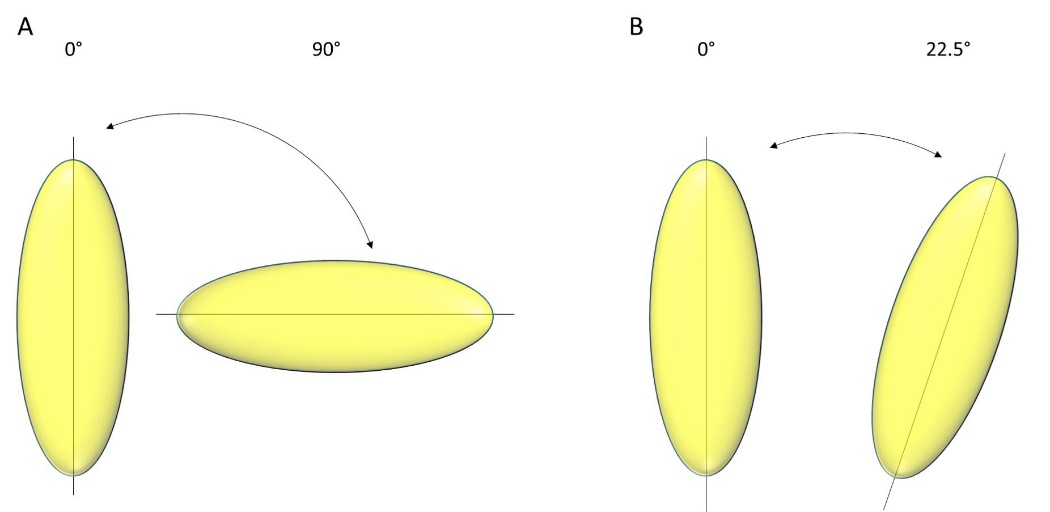


***Figure S5: In silico models of osteocyte lacunae in different bone types.*** *(A) Case of woven bone with random orientation of osteocyte lacunae (rotations between 0˚ and 90˚* *around the x-, y-, and z-axis) and (B) case of parallel-fibred bone with longitudinally aligned osteocyte lacunae (rotation between 0˚ and 22.5˚ around the x-, y-, and z-axis).*


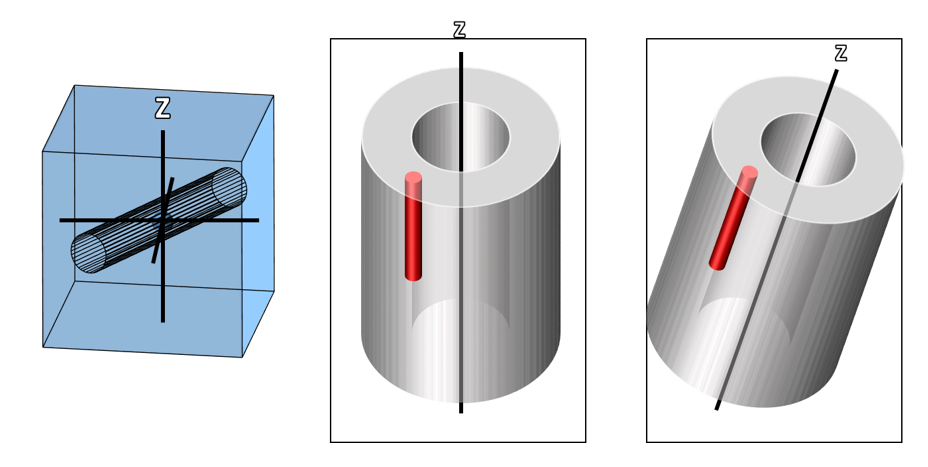


***Figure S6: The longitudinal angle ω was estimated relative to the main longitudinal axis of the bone.*** *(A) For in silico test datasets, the shape was created in a cubic domain and the ‘longitudinal bone axis’ was fixed as parallel with the z-axis of the domain. (B) In experimental datasets, the angle was corrected and estimated relative to the longitudinal bone axis (z-axis) rather than the z-axis of the dataset.*
